# Talkin’ about a revolution: integrating parents of children with Down syndrome as experts-by-experience in pediatric outpatient care

**DOI:** 10.1007/s00431-025-06532-8

**Published:** 2025-10-14

**Authors:** V. J. T. Peters, A. M. de Hoog, H. B. M. van Gameren-Oosterom, J. P. de Winter, T. Eulderink, L. A. Bok

**Affiliations:** 1 Department of Information Systems and Operations Management, Tilburg University, Tilburg, the Netherlands; 2 Department of Pediatrics, Groene Hart Ziekenhuis, Gouda, the Netherlands; 3 Department of Pediatrics, Spaarne Gasthuis, Haarlem, Hoofddorp the Netherlands; 4 Leuven Child and Health Institute, KU Leuven, Leuven, Belgium; 5 Department of Development and Regeneration, KU Leuven, Leuven, Belgium; 6 Department of Pediatrics, Máxima Medisch Centrum, Veldhoven, the Netherlands

**Keywords:** Experts-by-experience, Quality of care, Down syndrome

## Abstract

This study explores the role of parents of children with Down syndrome as experts-by-experience in pediatric outpatient care, a setting where their integration remains understudied, despite growing evidence of their value in other healthcare settings. We conducted a qualitative, exploratory interview study and took the outpatient care provision for children with Down syndrome as our setting. We conducted 18 interviews with healthcare professionals, parents of children with Down syndrome, and experts-by-experience. We utilized thematic analysis to analyze our data. We find that experts-by-experience act as a bridge between healthcare professionals and parents and improve the quality of care and life for children with Down syndrome and their families by offering support that extends beyond medical care into the social-emotional and practical aspects of daily living.

*Conclusion*: Experts-by-experience offer unique contributions to pediatric outpatient care. Their lived experience makes them valuable contributors to improve the quality of care and life. We call for every outpatient multidisciplinary team to integrate experts-by-experience as standard practice.

**Table:** 

**What is Known:** • *Experts-by-experience are increasingly recognized as valuable contributors in healthcare settings, offering unique perspectives that can enhance care provision. *
**What is New:** • *The involvement of parents as experts-by-experience in pediatric outpatient care improves the quality of care and quality of life for children and their parents.*

**Supplementary Information:**

The online version contains supplementary material available at 10.1007/s00431-025-06532-8.

## Introduction

Children and their parents are increasingly recognized as valuable partners in care provision, offering unique perspectives on how care should be delivered [1, 2]. They are the ones to share insights on what works best for them, ideally positioned to identify areas for improvement that can benefit themselves, their families, and their fellow patients. This is important given the diversity in the pediatric patient population and the global call for patient-centered care provision [3], meaning that care should be tailored to meet the complex care needs in individuals, such as children with epilepsy or children with coeliac disease [4]. One way to optimize the individual needs and preferences of pediatric patients is by utilizing the “experiential knowledge” of children and/or their parents. Experiential knowledge is defined as “having knowledge and insights developed through reflection on and analysis of explicit, concrete personal experiences, acquired by the person themselves and by others” [5]. Incorporating such expertise in healthcare reflects the principles of co-production, where professionals and patients collaborate as partners to design and deliver care together [6]. When someone can transfer their experiential knowledge to others, they are considered an expert-by-experience (EbE) [7], individuals who have direct experience with health and social care services, either as users or as caregivers for those who do.

EbE are increasingly recognized as valuable contributors to healthcare, offering unique perspectives that can enhance care provision [8]. Their lived experience allows them to engage with professionals on equal footing [9]. Their contributions are gaining recognition in care settings such as elderly care, intellectual disability care, and mental healthcare [10–12]. EbE can provide patients and families deeper insights into care provision, leading to improved experiences and outcomes [13], while also benefiting personally from their involvement [9]. However, integrating EbE in pediatric outpatient settings has not been investigated, which is striking because EbE could serve a role as volunteers who collaborate with healthcare professionals by drawing on their own past experience with similar situations. Therefore, this study aims to explore the role of EbE in pediatric outpatient care to improve quality of care.

## Methods

### Study design

The consolidated criteria for reporting qualitative research (COREQ) [14] were used as a guideline for this qualitative, exploratory interview study (Appendix 1). Ethical approval for this study was obtained from the Ethics Review Board of Tilburg University [EC-2017.60t]. The research was conducted in accordance with the Declaration of Helsinki.

### Recruitment and sample

This study was conducted at a pediatric outpatient clinic of a teaching/general hospital in the Netherlands. This clinic organizes multidisciplinary team appointments for children with Down syndrome (DS), a so-called Downteam [15], including consultations with an audiologist, dietitian, ENT doctor, EbE (parent of a child with DS), eye doctor, pediatrician, physiotherapist, and speech therapist. During a Downteam visit, children with DS and their families meet these specialists consecutively on the same day [16]. The Downteam convenes before and after to jointly discuss each child’s findings; EbE contribute the lived-experience perspective alongside the medical perspective from healthcare professionals. Recruitment of participants was carried out by a pediatrician, the coordinator of the Downteam, based on purposive sampling. This sampling technique involves identifying and selecting individuals that are especially experienced in the phenomenon of interest [17]. Using face-to-face request, the pediatrician selected three types of relevant participants: (1) EbE who participate(d) in the Downteam, (2) parents of children with DS who receive(d) care at the Downteam, and (3) healthcare professionals who are members of the Downteam. A total of 18 interviews were conducted: four interviews with EbE, seven interviews with parents of children with DS receiving care, and seven interviews with healthcare professionals (Table 1). Verbal and written informed consent was obtained from each participant prior to the interview. To minimize potential power imbalances, interviews were conducted by an independent researcher not involved in the care process, with confidentiality assured and participants reminded that they could decline questions or withdraw at any time.

**Table 1:** Respondent characteristics

Respondent	Profession	Gender	Age	Experience with DS care
Respondent 1	Expert-by-experience	F	50	Daughter with DS (18 years old)
Respondent 2	Expert-by-experience	F	63	Daughter with DS (29 years old)
Respondent 3	Expert-by-experience	F	54	Son with DS (16 years old)
Respondent 4	Expert-by-experience	F	62	Daughter with DS (30 years old)
Respondent 5	Dietician	F	51	13 years active in outpatient clinic
Respondent 6	Audiology assistant	F	49	9 years active in outpatient clinic
Respondent 7	Secretary	F	43	5 years active in outpatient clinic
Respondent 8	Pediatrician	M	66	20 years active in outpatient clinic
Respondent 9	ENT doctor	F	37	2 years active in outpatient clinic
Respondent 10	Physiotherapist	F	51	13 years active in outpatient clinic
Respondent 11	Speech therapist	F	48	13 years active in outpatient clinic
Respondent 12	Parent	F	48	Daughter with DS (16 years old)
Respondent 13	Parent	F	54	Son with DS (21 years old)
Respondent 14	Parent	M	61	Son with DS (22 years old)
Respondent 15	Parent	F	41	Daughter with DS (13 years old)
Respondent 16	Parent	F	45	Son with DS (16 years old)
Respondent 17	Parent	F	43	Son with DS (9 years old)
Respondent 18	Parent	F	48	Daughter with DS (16 years old)

### Data collection

We conducted 18 semi-structured interviews either in-person or via Microsoft Teams in October and November 2024. The semi-structured interviews lasted 20 to 70 min and involved open-ended questions regarding the perceived experience, collaboration, added value, and concerns with EbE in pediatric outpatient care of DS patients. An interview topic guide was used for the interviews (Appendix 2–4); the same topics were discussed with all respondents, yet questions were adapted to the perspective of the type of respondent. Each interview was audio-recorded and transcribed verbatim. Respondents were asked to review their own transcript to improve the reliability of our interpretations; no significant corrections were made to the transcripts. This was done to check for accuracy and resonance with the respondents [18].

### Data analysis

To analyze our interview data, we conducted a thematic analysis [19]. Our coding scheme, including our main themes and subthemes, is presented in Appendix 5. LH and VP individually coded all transcripts using Atlas.ti software. No new themes emerged after 15 interviews, indicating data saturation was achieved.

## Results

### Perspectives on the role of experts-by-experience

The respondents highlighted both benefits and complexities of the involvement of EbE in pediatric outpatient care. Parents generally had a positive attitude towards EbE, as they found it valuable to talk with someone who had lived through similar experiences. Many parents expressed comfort in sharing their stories, appreciating the informal and equal nature of the conversations, which allowed for practical advice and emotional support that often went beyond the scope of healthcare professionals. As one parent expressed: “Yes, definitely, especially to be able to brainstorm together, exchange ideas, and still have that sense of emotion, recognition, and understanding, which was really important. You could talk on the same level, and there was nothing better than learning from each other and discovering.” However, a minority of the parents remained reserved and did not feel the need to share personal matters, which the EbE respected and acknowledged.

Healthcare professionals universally highlighted the contribution of EbE, emphasizing their indispensable role within the care team. The professionals considered the EbE’s experiential expertise complementary to their own medical knowledge, offering unique insights into how parents perceive challenges and how certain healthcare decisions affect their daily lives. This recognition reinforces the importance of the EbE in providing a well-rounded approach to care, as they offer valuable perspectives that might otherwise be overlooked.

The EbE themselves reported a positive experience working within outpatient care. They valued the open, respectful, and constructive atmosphere of the care team, which fostered collaboration and mutual respect. While they acknowledged that the primary focus of the team was medical care, they appreciated the opportunity to address the socio-emotional dimensions of care for children with DS, contributing to an integral approach that supported both the medical and emotional well-being of the children and their families.

### Personal characteristics experts-by-experience

The interviewees expressed various personal characteristics that were considered important for an EbE in outpatient care provision: (1) utilizing lived experience, (2) being able to clearly communicate and listen, (3) being open-minded and showing empathy, and (4) maintaining role awareness and distance.

### Utilizing lived experience

Utilizing lived experience is central to the role of EbE in supporting parents of children with DS. Respondents emphasized that a strong foundation of both personal experience and knowledge is essential for EbE to provide meaningful guidance to parents and colleagues. Their own journeys of raising a child with DS equip them with deep insights into medical, developmental, and practical aspects of care (which school, which daycare, which playground, etc.): from early interventions like speech and physical therapy to navigating daily challenges throughout different life stages.

EbE actively deepens their knowledge to raise their kid with DS through affiliations with organizations like the Dutch Down Syndrome Foundation, gaining access to up-to-date information, attending events, and facilitating peer exchange. Many pursue learning by attending courses, workshops, and utilizing information they obtain from parents to stay informed beyond their own situations. Some even develop educational materials and online resources, extending their impact. To illustrate: “And when parents come to our clinic and say: ‘My child goes to this and that soccer club, and it is really great,’ we [EbE] make a note of it, those soccer clubs. This is how we gather our knowledge as well”—EbE 2.

### Communication and listening skills

Many respondents underline the importance of strong, sensitive, and adaptive communication and listening skills. EbE must be more than just good conversationalists—they need to actively listen, read between the lines, and ask the right follow-up questions to understand the needs of parents. Striking a balance between sharing personal experiences and giving space for parents to express their thoughts is crucial. Their goal is not to dominate the conversation but to respond appropriately to what the parent is seeking—whether that be practical tips, emotional reassurance, or simply a listening ear. Parents expect that EbE guide conversations gently, using subtle questions that allow parents to open up at their own pace, without feeling pressured or overwhelmed. This two-way interaction is key to creating trust and ensuring that the conversation remains meaningful and parent-centered: “The question someone asks, even if they cannot express it clearly, trying to analyze where the problem lies, where it comes from. And being socially skilled enough to ask the right questions to bring the problem to light”—Parent 2.

### Being open-minded and showing empathy

Openness and empathy are reported as essential characteristics for EbE to build meaningful and supportive relationships with (parents of) children with DS. EbE must be open to a wide range of perspectives without judgment, respecting different parenting choices, and remaining neutral to create a safe, respectful space for open dialogue. This includes being receptive to diverse perspectives for a child’s future and offering insights without imposing personal beliefs: “And what I learned the most there is not to judge other people. I used to always be inclined to view things from my own situation and perspective. So, I learned not to have an opinion about it [raising a kid], not to reflect it onto myself, but to accept that they do it the way they do. And that it is not right or wrong”—EbE 3. Empathy is equally crucial. EbE are expected to be emotionally aware, genuinely engaged, and capable of offering reassurance that puts parents at ease. Most respondents agreed that empathy is a strength among EbE; however, one parent noted that in some instances, this empathy could feel superficial.

### Maintaining role awareness and distance

Both EbE and healthcare professionals emphasized the importance of understanding the boundaries of the EbE role. Role awareness involves recognizing what is within the scope of their support and clearly communicating what they can and cannot offer. For example, when parents require deeper psychological support, EbE should acknowledge their limitations and advise them to go to professionals such as psychologists or social workers. Equally important is the ability to maintain emotional distance. They must avoid becoming overly emotionally involved or taking on the parents’ burdens as their own, as mentioned by the pediatrician: “[Name of EbE] could better maintain distance, meaning they could listen well, acknowledge, and reflect, but not get emotionally carried away.” This balance allows them to remain present and compassionate while also preserving the objectivity and professionalism necessary to fulfill their role within the care team.

### Organizational prerequisites

The EbE participate voluntarily in outpatient care provision and do not receive formal support from the hospital. They have taken the role of EbE based on their own initiative and personal experience and have signed a contractual appointment as a volunteer with the hospital. Role clarity and (formal) training have been mentioned during the interviews as organizational prerequisites to fulfill their roles as EbE.

### Role clarity

Respondents mentioned that ambiguity sometimes exists regarding the roles and responsibilities of different team members, including EbE. Some EbE expressed a need to better understand in advance which topics healthcare professionals cover in conversations with parents, to avoid overlap. Similarly, healthcare professionals also indicated a need for clearer communication about the EbE’s role. Understanding the specific contributions of EbE, including their lived experience and connections with relevant organizations, would strengthen collaboration and integration within the care team. As illustrated by an ENT doctor: “Even if it is just for five minutes or if they [EbE] send a short email with their details, such as their experience, how they work, and which patient organization they are connected to, I would have immediately gone along with it. It became clearer as time went on, but it would have been more convenient if it had been done upfront.” Role clarity is also essential from the perspective of parents. Several parents reported being initially unaware of the kind of support EbE could offer, leading to missed opportunities for timely advice or emotional support. One parent shared that not understanding the EbE’s role early on meant missing out on help during a challenging period with their child’s behavior.

### Lack of (formal) training

The lack of (formal) training for EbE was noted as a potential area for improvement, though perceptions of its importance varied. Most EbE themselves did not view the lack of training as a major issue, with only one expressing a desire for such support. However, some healthcare professionals saw value in offering targeted training, particularly in communication techniques, to enhance their role. Parents emphasized the need for EbE to be well-informed about practical matters such as local and national regulations, available subsidies, and relevant resources for children with DS. They suggested the implementation of regular workshops or a learning network to keep EbE up to date with current developments. Such initiatives would allow EbE to complement their personal experiences with up-to-date knowledge, better equipping them to guide families and connect them with regional opportunities and support systems: “And I can imagine that the information they [EbE] gave me five years ago is no longer accurate. So, in that sense, a course would be useful, but also knowing what regulations exist and keeping that information up to date, so they can inform people about it”—Parent 2.

### Quality of care

All respondents expressed that EbE adds to the quality of pediatric outpatient care. Both parents and EbE acknowledged the role of EbE in improving access to care for children with DS. EbE helps parents connect with the “right” (healthcare) professionals, drawing on their own personal experiences and offering valuable insights in areas like speech therapy, education, and support programs. This guidance empowers parents to navigate the healthcare system, make informed decisions that align with their child’s unique needs, and fully utilize available resources.

EbE also contribute to patient-centered care provision by acting as a bridge between healthcare professionals and parents. Parents often feel more comfortable expressing their concerns to EbE, as they trust them due to their shared (lived) experiences and understanding of their situation. This trust breaks down barriers that might otherwise prevent parents from voicing important issues. EbE, in turn, communicate these concerns to the care team, enabling professionals to address the specific needs of each child, ensuring that care plans are tailored to the individual. For example, one EbE explained how she raised concerns about a father struggling to accept his child with DS: “I also had a mother where the father could not accept their child. He just left the child [and mother] alone. And it is really nice that you can discuss all of this later with the pediatrician, saying, we really need to set up something here, we need to send someone [home care provider]to the family to see how things are going”—EbE 3. This illustrates how EbE can signal family issues that may otherwise remain unnoticed, resulting in tailored care. Furthermore, EbE help parents prepare for conversations with healthcare professionals, enabling them to approach discussions with more confidence and clarity.

### Quality of life

Not only do EbE play a valuable role in enhancing the quality of care, but they also improve the quality of life for children with DS and their families by offering support that extends beyond medical care into the social-emotional and practical aspects of daily living. They guide parents in promoting their child’s social and emotional development—such as building friendships and encouraging participation in everyday activities—helping families overcome fears or uncertainties. For instance, one mother—although the doctors had no doubts she initially hesitated to let her child with a pacemaker play outside—gained the confidence to do so with EbE’s encouragement, enabling her child to engage socially and physically in a safe way. EbE also help parents recognize and address developmental needs, such as teaching children ergonomic skills like eating independently, thereby improving the child’s autonomy and self-esteem. EbE also offer practical tips on accessing resources like respite care, swimming lessons, playgrounds, transportation to school, and additional support. This eases the burden on parents, giving them more time to rest, recharge, and enjoy family life: “So, if you can be relieved, whether it's through opportunities like overnight stays or respite care, or help in the classroom, help at home, transportation, or whatever. The point is that [EbE] they always find ways to facilitate things around you to relieve you”—Parent 2.

## Discussion

This study is, to our knowledge, the first to explore the role of parents as EbE in pediatric outpatient care, and potentially in hospital care more broadly. The findings provide novel insights into the involvement of EbE in Downteams in the Netherlands and highlight both the added value and challenges associated with their involvement. All respondents were positive about the role of EbE in outpatient care. They described EbE as parents who actively deepen their knowledge to support their own child’s development and quality of life, and whose involvement illustrates how co-production can take shape in practice [6]. Key personal EbE characteristics were identified, including the ability to use lived experience effectively, strong communication and listening skills, open-mindedness, empathy, role awareness, and the ability to maintain professional distance. These findings highlight that not everyone is suited to be an EbE and that careful selection and training are beneficial, which raises questions about the feasibility of EbE involvement. Recruiting a sufficient number of volunteers with condition-specific expertise is not as easy as it may appear given that most EbE are volunteers with ongoing caregiving responsibilities for their own child. Incorporating EbE into outpatient care should therefore not be taken lightly; it requires careful planning, investment, and appropriate support, and must avoid the assumption that EbE can simply be added without addressing these considerations. If done right, EbE involvement enhances outpatient team’s credibility by strengthening the trustworthiness, quality, and expected outcome of the (medical) advice given.

Our identified benefits of EbE align with findings from other care settings like elderly care, mental care, and intellectual disability care [10–12]. EbE contribute to patient-centered care provision by acting as a bridge between healthcare professionals and parents/relatives. Also, EbE help parents prepare for conversations with healthcare professionals, enabling them to approach discussions with more confidence and clarity [20]. Additionally, they improve the quality of life for children with DS and their families by offering support that extends beyond medical care into the social-emotional and practical aspects of daily living. On top of that, in our study, respondents described EbE as indispensable team members who provide unique emotional and informational parental support, bridge communication gaps between professionals and parents, and contribute to shared decision-making and empowerment. Notably, the quote from one healthcare professional who claimed they “would not want to run the clinic without them” strongly underscores the perceived value of EbE in the pediatric context.

While the DS outpatient setting is characterized by a chronic and multidisciplinary care trajectory, this is not unique to DS. Similar trajectories exist in other pediatric conditions, and we believe that these too could benefit from the involvement of EbE with condition-specific expertise. This may enhance the relevance of EbE, who can offer lived-experience insights not only on acute care issues but also on long-term developmental, educational, and social challenges. The DS context may prove particularly meaningful for EbE involvement due to the high emotional burden, the diversity of care needs, and the complexity of navigating the care journey across primary and secondary care providers [4]. In this way, EbE contribute to integral care delivery that is essential in the multifaceted care for children with Down syndrome.

Several organizational prerequisites remained underdeveloped. The lack of (formal) training and unclear role definitions were recurrent themes in the data, similar to previous research indicating that EbE felt unprepared for their role [11]. Our findings suggest that training in communication skills, knowledge of relevant (local) legislation and regulations, managing emotions, and clarifying boundaries could enable EbE to feel better prepared and support parents more effectively. EbE and healthcare professionals emphasized the need for role clarity and support to improve the involvement of EbE in pediatric outpatient care. As one EbE noted, participation in the multidisciplinary team meetings before and after the Downteam takes place, and aligning with professional roles, enhanced both their confidence and effectiveness.

## Limitations and future research

Several limitations must be acknowledged. First, this study was conducted in a single Dutch hospital with close to 20 years of experience involving EbE, which fostered trust and clear routines at the outpatient setting. Such conditions may not exist in hospitals new to EbE involvement. As such, the results may reflect a particularly mature model of EbE involvement. Future research should explore EbE involvement in pediatric settings at other (Dutch) hospitals, including those without such a longstanding tradition, to assess the generalizability of our findings. Second, we deliberately focused on care provision for children with DS. While the DS context has specific characteristics that may facilitate EbE involvement, such as lifelong support needs, it is important to uncover whether similar benefits are found in other pediatric populations. Possible target groups for future research include children with intellectual disabilities more generally or other chronic care populations such as those with cystic fibrosis, cerebral palsy, Duchenne, or diabetes.

## Conclusion

We explored the role of parents as EbE in pediatric outpatient care provision, specifically for children with DS in the Netherlands. We focused on how their lived experience can enhance the quality of care for pediatric patients. We find that EbE act as a bridge between healthcare professionals and parents and improve the quality of life for children with DS and their families by offering support that extends beyond medical care into the social-emotional and practical aspects of daily living.

## Supplementary Information

Below is the link to the electronic supplementary material.

ESM 1 (DOCX 19.9 KB)

ESM 2 (DOCX 17.3 KB)

ESM 3 (DOCX 16.8 KB)

ESM 4 (DOCX 16.3 KB)

ESM 5 (DOCX 17.8 KB)

## Data Availability

The data that support the findings of this study are available from Tilburg University, but restrictions apply to the availability of these data. The data are, however, available from the authors upon reasonable request and with the permission of the Ethics Review Board of Tilburg University.
