# Supplementary material for: Talkin’ about a revolution: integrating parents of children with Down syndrome as experts-by-experience in pediatric outpatient care
Source: Eur J Pediatr. 2025 Oct 14;184(11):689. doi: 10.1007/s00431-025-06532-8 (PMC12521312; doi:10.1007/s00431-025-06532-8)
Supplement: Supplementary file 1 — (DOCX 19.9 KB) [file 431_2025_6532_MOESM1_ESM.docx]

**Appendix 1. A 32-item checklist for reporting qualitative studies (COREQ)**

| **Item** | **Description** | **Check** |
| --- | --- | --- |
| **Domain 1: Research team and reflexivity** | | |
| *Personal characteristics* | | |
| 1. Interviewer | Which author/s conducted the interviews and/or observations? | Author 2 |
| 2. Credentials | What were the researcher’s credentials? | Author 1: Dr.  Author 2: MSc  Author 3: Dr.  Author 4: Prof. dr.  Author 5: N/A  Author 6: Dr. |
| 3. Occupation | What was the occupation at the time of the study? | Author 1: Assistant professor  Author 2: Master’s student  Author 3: Pediatrician  Author 4: Pediatrician  Author 5: Expert-by-experience  Author 6: Pediatrician |
| 4. Gender | Was the researcher male or female? | Author 1: Male  Author 2: Female  Author 3: Female  Author 4: Male  Author 5: Female  Author 6: Male |
| 5. Experience and training | What experience or training did the researcher have? | Courses on Qualitative Research Methods at Tilburg University and  previous experience during thesis writing. |
| *Relationship with participants* | | |
| 6. Relationship established | Was a relationship established prior to study commencement? | A professional relationship was established with the healthcare professionals, based on previous collaborations, ahead of the research. |
| 7. Participant knowledge of the researcher | What did the participants know about the researcher? | The personal interest of the researchers and purpose of the study was explained before the data collection started. |
| 8. Researcher characteristics | What characteristics were reported about the researcher? | Interest in research topic, occupation, reason for research |
| **Domain 2: Study design** | | |
| *Theoretical framework* | | |
| 9. Methodological orientation and Theory | What methodological orientation was stated to underpin the study? | Thematic analysis |
| *Participant selection* | | |
| 10. Sampling | How were participants selected? | Purposive sampling |
| 11. Method of approach | How were participants approached? | Telephone, face-to-face, e-mail |
| 12. Sample size | How many participants were in the study? | 18 |
| 13. Non-participation | How many people refused to participate or dropped out? Reasons? | 1; not available due to time investment |
| *Setting* | | |
| 14. Setting of data collection | Where was the data collected? | In-person setting (home or work environment) and digital (Microsoft Teams) |
| 15. Presence of non-participants | Was anyone else present besides the participants and researchers? | No |
| 16. Description of sample | What are the important characteristics of the sample? | Gender, profession, role in outpatient care, age of child |
| *Data collection* | | |
| 17. Interview guide | Were questions, prompts, guides provided by the authors?  Was it pilot tested? | The topic list is added to the manuscript; the topic list was pilot tested and discussed within the study team. |
| 18. Repeat interviews | Were repeat interviews carried out? If yes, how many? | No repeat interviews were carried out. |
| 19. Audio recording | Did the research use audio recording to collect the data? | Yes |
| 20. Field notes | Were field notes made during and/or after the interview or observation? | Yes |
| 21. Duration | What was the duration of the interviews or observation? | Interviews: 20-75 minutes |
| 22. Data saturation | Was data saturation discussed? | Data saturation was discussed within the research team. |
| 23. Transcripts returned | Were transcripts returned to participants for comment and/or correction? | Transcripts were returned to participants. We received no comments and/or corrections. |
| **Domain 3: Analysis and findings** | | |
| *Data analysis* | | |
| 24. Number of data coders | How many data coders coded the data? | Two (Author 1 and Author 2) |
| 25. Description of the coding list | Did authors provide a description of the coding list? | The coding list is described in the Methods section. |
| 26. Derivation of themes | Were themes identified in advance or derived from the data? | Themes were identified in advance, but we also made use of a code called ‘other’ in which relevant other themes were categorized. |
| 27. Software | What software, if applicable, was used to manage the data? | Microsoft Word, Atlas.ti |
| 28. Participant checking | Did participants provide feedback on the findings? | We asked the participants to reflect on the findings of the study and received no comments or corrections. |
| *Reporting* | | |
| 29. Quotations presented | Were participant quotations presented to illustrate the themes/findings? Was each quotation identified? | We used various quotations from our participants to illustrate our findings. |
| 30. Data and findings consistent | Was there consistency between the data presented and the findings? | We present an analytic story where we highlight the key themes of the study. |
| 31. Clarity of major themes | Were major themes clearly presented in the findings? | We present major themes (characteristics of experts-by-experience) in the Results section. |
| 32. Clarity of minor themes | Is there a description of diverse cases or discussion of minor themes? | The minor themes support the characteristics of experts-by-experience) and are presented in the Results section of the study. |
