# Supplementary material for: Talkin’ about a revolution: integrating parents of children with Down syndrome as experts-by-experience in pediatric outpatient care
Source: Eur J Pediatr. 2025 Oct 14;184(11):689. doi: 10.1007/s00431-025-06532-8 (PMC12521312; doi:10.1007/s00431-025-06532-8)
Supplement: Supplementary file 2 — (DOCX 17.3 KB) [file 431_2025_6532_MOESM2_ESM.docx]

**Appendix 2. Interview topic guide Expert-by-Experience**

**General introduction**

1. What is your name, age, and experience with children with Down syndrome?
2. What motivated you to become an expert-by-experience in the care for children with Down syndrome?

**Role and activities**

1. How often do you have conversations with parents, and how long do these usually last?
2. Can you describe how these conversations typically go?
3. Can you describe the kind of support you provide to parents of children with Down syndrome?

**Experience with parents**

1. How do you experience the attitude of parents towards you as an expert-by-experience?
2. If parents are not open to a conversation, how do you handle that situation?
3. Can you give examples of situations where you noticed that your involvement made a difference for a parent?
4. Can you also give an example of a situation where you noticed you were unable to support a parent?
5. How do you build a relationship with the parents and the children, and what are the biggest challenges and successes in doing so?

**Support and personal/organizational characteristics**

1. What kind of support have you received to fulfill your role as an expert-by-experience within the Downteam?
2. What personal characteristics do you think are important for an expert-by-experience to be successful?
3. What organizational factors do you consider essential for an expert-by-experience to function effectively?

**Collaboration with healthcare professionals**

1. How do you experience the collaboration with other members of the Downteam, and what do you see as the biggest challenges in working within the team?
2. Can you describe your role within the Downteam? What are your main responsibilities in the team?

**Impact on quality of care**

1. How do you think your experiences and insights contribute to the quality of care for children with Down syndrome?

17. Do you have any suggestions for how the role of experts by experience could be improved within hospital care for children with Down syndrome?

18. Do you have any suggestions for improving collaboration between experts by experience and healthcare professionals?

**Closing**

1. Do you have any further questions or comments?
