# Supplementary material for: Talkin’ about a revolution: integrating parents of children with Down syndrome as experts-by-experience in pediatric outpatient care
Source: Eur J Pediatr. 2025 Oct 14;184(11):689. doi: 10.1007/s00431-025-06532-8 (PMC12521312; doi:10.1007/s00431-025-06532-8)
Supplement: Supplementary file 3 — (DOCX 16.8 KB) [file 431_2025_6532_MOESM3_ESM.docx]

**Appendix X. Interview topic guide parent of child with Down Syndrome**

**General introduction**

1. What is your name and age?
2. What has been your experience with hospital care for children with Down syndrome?
3. Had you heard of experts-by-experience before coming to the MMC, and what was your first impression?

**Support from experts-by-experience**

1. How often do you speak with the experts-by-experience, and how long do these conversations usually last?
2. How do these conversations go?
3. How do you experience the support provided by experts-by-experience in the care of your child?
4. Can you describe a specific situation in which an expert-by-experience helped or supported you?
5. Can you describe a specific situation in which an expert-by-experience was not able to help or support you?

**Personal and organizational characteristics**

1. What personal characteristics do you think an expert-by-experience should have to provide effective support?
2. Are there also organizational aspects that you think are important for the involvement of experts-by-experience?

**Challenges and value of experts-by-experience**

1. Do you experience any challenges in your contact with the experts-by-experience, and if so, what are they?
2. Which aspects of the support from experts-by-experience do you find most valuable, and which do you find least valuable?
3. How do you think the role of the expert-by-experience influences the quality of care for your child?

**Improvements and recommendations**

1. Do you have any suggestions for how the role of experts-by-experience could be improved within hospital care for children with Down syndrome?
2. If a conversation with an expert-by-experience were no longer offered as a standard part of the Downteam, would you still want to make use of this support?
3. Would you recommend the involvement of experts-by-experience to other parents of children with Down syndrome, and why?

**Closure**

1. Do you have any further questions or comments?
