# Supplementary material for: Talkin’ about a revolution: integrating parents of children with Down syndrome as experts-by-experience in pediatric outpatient care
Source: Eur J Pediatr. 2025 Oct 14;184(11):689. doi: 10.1007/s00431-025-06532-8 (PMC12521312; doi:10.1007/s00431-025-06532-8)
Supplement: Supplementary file 4 — (DOCX 16.3 KB) [file 431_2025_6532_MOESM4_ESM.docx]

**Appendix 4. Interview topic guide healthcare professional**

**General introduction**

1. What is your name, age, and role within the Downteam?
2. How long have you been working with children with Down syndrome?

**Communication and collaboration**

1. How and when do you encounter experts-by-experience during the care process for children with Down syndrome?
2. How does the communication between you and the experts-by-experience go?
3. What is your experience working with experts-by-experience in the care for children with Down syndrome?
4. What are the biggest challenges you experience when collaborating with experts-by-experience within the Downteam?

**Value and impact**

1. In your opinion, what value does an expert-by-experience add to the Downteam?
2. How does this impact the quality of care for children with Down syndrome?
3. Can you give an example of a situation in which the experiences of an expert-by-experience contributed positively to the care you provided?
4. What do you see as the advantages of involving experts-by-experience in supporting parents of children with Down syndrome, and what might be potential disadvantages?

**Characteristics and improvement**

1. What personal characteristics do you consider important for an expert-by-experience to be part of the Downteam?
2. In your opinion, are there also organizational factors that are important for the involvement of experts-by-experience in the Downteam?
3. Do you see any points for improvement or opportunities to further optimize the role of experts-by-experience within the Downteam?

**Closing**

1. Do you have any further questions or comments?
