# Supplementary material for: Talkin’ about a revolution: integrating parents of children with Down syndrome as experts-by-experience in pediatric outpatient care
Source: Eur J Pediatr. 2025 Oct 14;184(11):689. doi: 10.1007/s00431-025-06532-8 (PMC12521312; doi:10.1007/s00431-025-06532-8)
Supplement: Supplementary file 5 — (DOCX 17.8 KB) [file 431_2025_6532_MOESM5_ESM.docx]

Appendix X. Coding scheme

| **Main theme** | **Subtheme** | **Illustrative/Exemplary quote** |
| --- | --- | --- |
| Perspectives on the role of experts-by-experience | n/a | *"They [experts-by-experience] have contributed a lot to the team. If they were not there, it would not be complete. I think they hold 40% of the shares. I would not propose a team without EbE. I would not want to run the clinic without them. I would be very much opposed to that. I would find it ridiculous." –* Healthcare professional 1 |
| Personal characteristics | - Utilizing lived experience - Communication and listening skills - Being open minded and showing empathy - Maintaining role awareness and distance | *“Well, that you [expert-by-experience] need to be empathetic, able to reassure parents and make them feel at ease.” –* EbE 4  *“It sometimes happens that parents sit with us for half an hour, crying. And we talk for that half hour. But we do not give the illusion that we can solve it for them. At most, we can reassure parents by saying: We recognize this, because we have experienced it ourselves or we have heard it from other parents.”* – EbE 3 |
| Organizational prerequisites | - Role clarity - Lack of (formal) training | *"But whether they can offer any courses, communication training, or something like that from the hospital, because those are available. Something like that would be really beneficial." –* Healthcare professional 6  “*At least a conversation with the coordinator of the Downteam. And maybe even with all the disciplines to discuss what each one talks about during that half hour. So that you know as EbE.*” *–* EbE 1  *"It is really a shame that I could not fully take advantage of their role, which I think is very important, at that time. I did what I could, which I also really appreciated, by having someone listen for half an hour and just telling them how things were going. But I could have gotten more out of it." –* Parent 7 |
| Quality of care | n/a | *Sometimes, outside the clinic, I help people apply for a personal budget. Or I refer people by saying, 'You need to call this and that person,' and then I give them the email address and phone number. And they will help you with that question. Not everyone is skilled enough to find all that on their own."* – Expert-by-experience 2  *If I said, well, the speech therapist recommended doing a bit more speech therapy outside of school, they often had contacts because, of course, they had older children. They would often provide addresses, recommend someone, or say, 'Oh, you should definitely do that, it's really helpful because it worked well for us.'" –* Parent 5  *"Yes, if I look back at the phase when [daughter's name] at some point... when we were talking about contraception, I initially discussed it with an EbyE, and then I went to the gynecologist. And I thought, 'Oh yes, this is what she [gynecologist] says,' then you really get a standard story. Whereas if you actually go into that conversation prepared, you can also make informed choices." –* Parent 1 |
| Quality of life | n/a | *"The child then, because they did everything themselves, went to a medical daycare center. He learned to eat, learned basic skills. And now, he is a happy boy and doing great." –* EbE 3 |
